# Reprogramming of glutamine metabolism and its impact on immune response in the tumor microenvironment

**DOI:** 10.1186/s12964-022-00909-0

**Published:** 2022-07-27

**Authors:** Guofeng Ma, Zhilei Zhang, Peng Li, Zhao Zhang, Manqin Zeng, Zhijuan Liang, Dan Li, Liping Wang, Yuanbin Chen, Ye Liang, Haitao Niu

**Affiliations:** 1 Department of Urology, The Affiliated Hospital of Qingdao University, No. 16 Jiangsu Road, Qingdao, 266003 China; 2 Key Laboratory, Department of Urology and Andrology, The Affiliated Hospital of Qingdao University, Qingdao, 266003 China; 3 Department of Pathology, The Affiliated Hospital of Qingdao University, Qingdao, 266003 China

**Keywords:** Glutamine metabolism, Reprogramming, Immune response, Tumor microenvironment, Immunity, Glutamine metabolism inhibitors

## Abstract

Metabolic reprogramming and immune escape play a major role in tumorigenesis. Increasing number of studies have shown that reprogramming of glutamine metabolism is a putative determinant of the anti-tumor immune response in the tumor microenvironment (TME). Usually, the predatory uptake of glutamine by tumor cells in the TME results in the limited utilization of glutamine by immune cells and affects the anti-tumor immune response. The cell-programmed glutamine partitioning also affects the anti-tumor immune response. However, the reprogramming of glutamine metabolism in tumors modulates immune escape by regulating tumor PD-L1 expression. Likewise, the reprogramming of glutamine metabolism in the immune cells also affects their immune function. Additionally, different types of glutamine metabolism inhibitors extensively regulate the immune cells in the TME while suppressing tumor cell proliferation. Herein, we discuss how metabolic reprogramming of tumor and immune cells regulates anti-tumor immune responses, as well as functional changes in different immune cells in the context of targeting tumor glutamine metabolism, which can better explain the potential of targeting glutamine metabolism in combination with immunotherapy for cancer.

**Video abstract**

**Supplementary Information:**

The online version contains supplementary material available at 10.1186/s12964-022-00909-0.

## Introduction

The onset and development of tumors are dependent on the impairment in the ecological balance of the body’s microenvironment, wherein metabolic reprogramming and the body’s immune response play important roles. Metabolic reprogramming and immune escape are considered to be the main characteristics of malignant tumors, which can be used to diagnose, detect and treat tumors [1, 2].In the past few decades, the Warburg effect has been considered as a typical example of metabolic reprogramming, wherein the tumor cells still rely on the conversion of glucose to lactate for energy needs under aerobic conditions, which is crucial for tumor initiation and progression [3, 4]. In addition to the key role of glucose, glutamine is also known to play an important role in tumorigenesis. As an important part of metabolic reprogramming in the tumors, reprogramming of glutamine metabolism is considered to have pleiotropic effects on cellular functions, such as macromolecule synthesis, energy generation, mTOR activation, maintenance of reactive oxygen species (ROS) balance and anti-tumor acidic microenvironment. Meanwhile, glutamine transporter mutants have been shown to promote metabolic reprogramming of tumors [5–7]. Recent studies have shown that glutamine is also an important raw material for the immune system, and plays a major role in regulating lymphocyte functions such as, the release of secretory factors; proliferation; and their general maintenance [8].

The body’s immune response involves a complex and dynamic biological network, including the innate and adaptive immune response, which play an important role in the onset and development of tumors. The innate immune response consists of many immune and non-immune cells, mainly including monocytes, macrophages, neutrophils, eosinophils, basophils, natural killer cells (NK cells), dendritic cells (DCs), platelets, epithelial cells, and fibroblasts. These cells together constitute an important barrier to prevent pathogens from infecting the body and help maintain the body’s homeostasis [9]. Adaptive immunity involves tightly regulated interactions between T and B lymphocytes and antigen-presenting cells, which promote the activation of pathogen-specific immune responses, the generation of immune memory, and the regulation of body’s homeostasis [10]. In fact, immune cells can sense various signal changes in the TME and turn on specific immune functions in response to those stimuli. Changes in nutrients and metabolites such as lactic acid, glucose and glutamine, in the TME, can affect the function of immune cells. Competition between tumor cells and immune cells in the TME can also affect the function of immune cells [11–19]. There is increasing number of studies reporting that glutamine affects the function of immune cells in the TME through multiple pathways, suggesting that intervention of glutamine metabolism may improve the effectiveness of anti-tumor immunotherapy.

In this review, we discuss the reprogramming of glutamine metabolism in tumor cells and immune cells within the TME, and the crosstalk between these cells. We also discuss how glutamine metabolism affects the biological changes in the tumor and immune cells, and affects the immune response. Finally, we also discuss the impact of glutamine metabolism inhibitors on the immune response.

## Overview of glutamine metabolism in tumor and immune cells

### Glutamine metabolism in tumor cells

Glutamine is the most abundant and widely used amino acid in the human body, which is an important source of nitrogen, and the respiratory fuel for tumor cells. It is an indispensable source of energy for maintaining tumor survival and progression [20]. It is known that some tumor cells consume a large amount of glutamine to meet their own metabolic needs. Tumor cells transport glutamine into cells through specific transporters (such as solute carrier family 1 neutral amino acid transporter member 5, SLC1A5; also known as alanine, serine, cysteine-preferring transporter 2, ASCT2), and then convert it into glutamate under the action of glutaminase (GLS), and further convert it into α-ketoglutarate (α-KG), which enters the Tricarboxylic Acid cycle (TCA) and participates in the onset, development and dissemination of tumors [21, 22]. For example, glutamine metabolites in tumor cells provides energy for tumor progression after entering the TCA cycle [6]. Glutaminolysis generates raw materials for the synthesis of macromolecular substances such as amino acids, nucleotides, fatty acids and hexosamines required by the tumor cells [23]. Glutamine contributes to the synthesis of uridine diphosphate-*N*-acetylglucosamine (UDP-GlcNAc), which is part of the hexosamine biosynthesis pathway (HBP) and is required for protein glycosylation and endoplasmic reticulum stress response in tumor cells [6]. Glutathione (GSH) and nicotinamide adenine dinucleotide phosphate (NADPH) synthesized by the glutamine metabolic pathway regulate the level of ROS in tumor cells to stabilize their redox homeostasis and ensure the survival of tumor cells [24, 25]. In addition, a complex relationship exists between tumor glutamine metabolism and autophagy. For example, ammonia produced by glutamine metabolism promotes autophagy in tumors [26, 27], and glutamine metabolism inhibits autophagy in tumor cells by activating the mTOR pathway [28].

The alterations in glutamine metabolism in tumor cells are an important outcome of the changes in the energy metabolism of tumor cells. Compared to the normal cells, the tumor cells express an abnormal level of regulatory molecules involved in glutamine metabolism. These regulatory molecules are often oncogenes or tumor suppressors that abnormally expressed in the tumors, and involved in tumor initiation and progression, such as *Myc, p53*, *Ras*, Hypoxia-inducible factor (HIF), Rho GTPase, etc. The above oncogenes or proteins may play a role in abnormal glutamine metabolism in the TME. For example, the amplification of *Myc* causes cellular addiction to glutamine, which may be related to the combined effects of *Myc* and glutamine transporter (such as SLC7A5 and SLC1A5) promoter elements, leading to enhanced glutamine uptake, which induces the activation of Lactate Dehydrogenase A (LDHA) and transports glucose-derived citrate out of the mitochondria, thereby increasing the requirement of glutamine [29–31]. As a suppressor gene, *TP53* is mutated or deleted in most tumors. On the one hand, *p53* promotes glutamine metabolism in tumor cells and makes them tolerant to the lack of glutamine by up-regulating GLS2, contributing to the survival of cancer cells. However, on the other hand, *p53* also increases the level of glutathione (GSH) in tumor cells, reduces ROS, and inhibits tumorigenesis [32–34]. The *Ras* oncogene promotes autophagy and glycolysis and regulates energy metabolism. *K-Ras* is known to make tumor cells more sensitive to glutamine deficiency, inhibit the expression of LDHA, and increase the expression of aspartate aminotransferase. Glutamine is the main carbon source for the TCA cycle when *Ras* is activated [21, 35–38]. HIF-1α and HIF-2α are highly expressed in most tumors [39]. In the human non-small cell lung cancer cell line A549, it was found that silencing HIF-1α expression reduced glutamine consumption in the tumor cells [40]. Furthermore, HIF-2α has been reported to enhance the activity of c-MYC, which in turn drives glutamine catabolism by regulating numerous genes including glutaminase [30, 41]. Rho GTPase regulates glutamine metabolism in a nuclear factor-kappa B (NF-κB)-dependent manner. For example, in human breast and lymphoid cancer cells, cancer cells that are dependent on Rho GTPase signaling have a higher GLS1 activity, which promotes tumor cell proliferation [42]. It can be appreciated that there is usually a reprogramming of glutamine metabolism in tumors, and tumor cells eventually choose the best mode of glutamine metabolism to adapt to their own survival and metabolic needs by regulating the mutation and expression of related genes.

### Glutamine metabolism in immune cells

Metabolic reprogramming plays a major role in: (a) the activation of immune cells; (b) the regulation of immune cell phenotype and function; (c) mounting a robust anti-tumor immune response [43–45]. Since immune cells play a key role in the host’s defense against infection and tumorigenesis, the metabolic changes in the immune cells have a major influence in regulating their pro-tumor or anti-tumor functions. These unique metabolic characteristics of immune cells are mainly reflected in the different metabolic patterns of immune cells during different cellular states, such as, quiescence, infection, or tumorigenesis [44, 46].For example, T cells exhibit completely different metabolic patterns depending on their activation state, for example, naive T cells have a minimum glycolytic rate and a minimum glutamine metabolism to maintain biosynthetic pathways for survival, whereas Teff cells have an increased glycolytic rate, and elevated glutamine metabolism, which enable the synthesis of proteins and nucleotides to meet the needs of rapidly proliferating tumor cells [47, 48]. For DCs, oxidative phosphorylation (OXPHOS) is the main source of energy at the resting state, while glycolysis is mainly used in the activated state [49].

Generally, the ratio of glutamine intake by immune cells is similar to or greater than that of glucose [50], and glutamine is converted to glutamate, alanine, and aspartate by partial oxidation to CO_2_ in immune cells. This unique transformation plays an important role in the functioning of immune cells. At the same time, the availability of glutamine largely determines the expression of certain genes in immune cells [51]. For example, during immune cell proliferation, glutamine induces the transcription of cell proliferation-related genes and promotes the proliferation of immune cells by activating proteins, such as ERK and JNK kinases and then acts on transcription factors, such as JNK and AP-1 [52]. Appropriate concentrations of glutamine promotes the expression of lymphocyte surface markers such as CD71, CD25, and CD45RO, and the production of cytokines such as IL-6, γ-interferon (IFN-γ), and TNF-α [53–56].Glutamine metabolism plays a major role in the activation of lymphocytes and is necessary for the differentiation of B lymphocytes into plasma cells and lymphoblasts. At the same time, glutamine is also necessary for T and B lymphocytes, for their proliferation, protein and antibody synthesis, and IL-2 production [57].Glutamine metabolism plays a key role in regulating macrophage activation, and the synthesis and secretion of pro-inflammatory cytokines, such as IL-1, TNF-α and IL-6. In addition, α-KG produced by glutamine metabolism promotes the differentiation of M2 macrophages [52, 58].Therefore, exploring the reprogramming of glutamine metabolism in immune cells and its impact on immune function will enable a better understanding of the mechanism by which glutamine metabolism regulates the TME and the body's immune response to tumors.

## Glutamine metabolic association between tumor cells and immune cells

### Glutamine competition

**Fig. 1:**
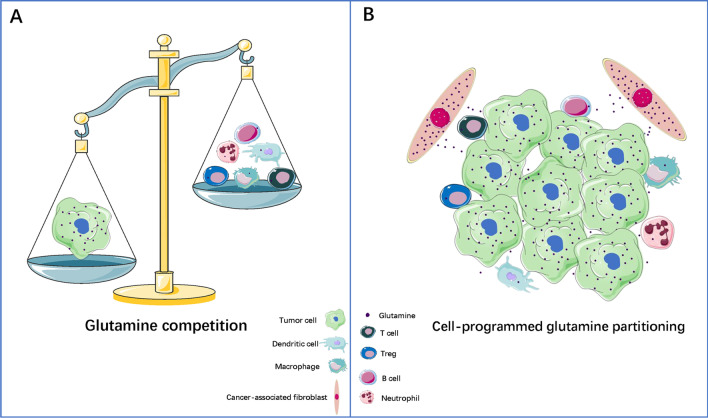
Association of glutamine metabolism with tumor cells and immune cells. The competition for glutamine between tumor cells and immune cells in the TME causes glutamine deficiency, which affects the function of immune cells, including macrophages, DCs, Treg cells, neutrophils, B cells and so on (**A**). Cell-programmed glutamine partitioning results in the highest consumption of glutamine by tumor cells in the TME. CAFs can up-regulate their glutamine synthesis, and complement glutamine depletion in the TME by secreting glutamine into the TME (**B**)

Like cancer cells, immune cells in the TME also undergo metabolic reprogramming [44]. Reprogramming of glucose metabolism is the most common phenomenon affecting energy metabolism in both tumor cells and immune cells. Both of these cells require glucose as an energy source, leading to a competition between them for glucose uptake in the TME [59–61]. Similarly, reprogramming of glutamine metabolism is also critical for the survival of tumor and immune cells, and competition for glutamine uptake also exists between these cells in the TME. For example, in glutamine-addicted clear cell renal cell carcinomas, the competitive consumption of glutamine by tumor cells results in local deprivation of extracellular glutamine, which activates HIF-1α and induces tumor-infiltrating macrophages to secrete IL-23. IL-23 further promotes the proliferation and activation of Treg cells, thereby suppressing the anti-tumor activity of Teff cells [62]. In triple-negative breast cancer (TNBC), studies have demonstrated that tumor cells competitively prey on glutamine in the TME, resulting in the limited availability of glutamine for tumor-infiltrating T lymphocytes, which affects their anti-tumor immune responses. Consistently, in the GLS-deficient mouse tumor model, the increased concentration of glutamine in the TME due to restricted glutamine utilization by tumor cells leads to elevated levels of glutamine available to tumor-infiltrating T lymphocytes, thereby enhancing its anti-tumor activity [17]. Mechanistically, the activation of the MAPK/ERK pathway plays a major role in promoting competition between tumor cells and T cells for glutamine uptake. Activation of the MAPK/ERK pathway not only up-regulates glutamine uptake by T cells, but also up-regulates glutamine uptake by tumor cells. Therefore, the differential expression of MAPK/ERK pathway-related proteins in the tumor cells and T cells may determine the cellular fate of competition between them for glutamine [63, 64]. In fact, a “glutamine steal” hypothesis has been proposed, which suggests that the selective blocking of glutamine metabolism in tumor cells could eliminate the metabolic competition for glutamine in the TME, while releasing glutamine for use by immune cells, so as to enhance anti-tumor immune response [17, 65] (Fig. 1A).

### Cell-programmed glutamine partitioning

Despite the growing evidence related to the competition for nutrients between tumor cells and immune cells in the TME, it is still unclear whether the dysregulation of immune cells metabolism and function in the TME arises due to cell-intrinsic programming or competition with cancer cells for the limited nutrients. Recent research has revealed significant differences in the uptake of glucose and glutamine by different cell subsets in the TME. Cancer cells are known to consume the highest amount of glutamine, while immune cells consume the most amount of glucose. This unique nutrient distribution is mainly regulated by the mTORC1 signaling pathway and the expression of genes related to the metabolism of glucose and glutamine. Thus, cell-intrinsic programs drive immune cells and cancer cells to preferentially consume glucose and glutamine, respectively [66].Similarly, there are cell-intrinsic programs in stromal cells in the TME, which regulate the local sources of glutamine to compensate for the glutamine depletion in the TME. For example, cancer-associated fibroblasts (CAFs) are usually in a state of metabolic symbiosis with cancer cells, and compared to the normal fibroblasts, glutamine synthesis is up-regulated in CAFs, and is accompanied by glutamine secretion to supplement the concentration of glutamine in the TME. Thus, co-culture with CAFs rescues cancer cell growth in glutamine-deficient TME as compared to co-culture with normal fibroblasts. At the same time, selective abrogation of glutamine anabolism in vivo in the CAFs has been shown to inhibit ovarian tumor growth in mice [67] (Fig. 1B).

## Effects of glutamine metabolism on immune response

### Metabolic reprogramming of glutamine metabolism in tumor cells and its impact on immune response

**Fig. 2:**
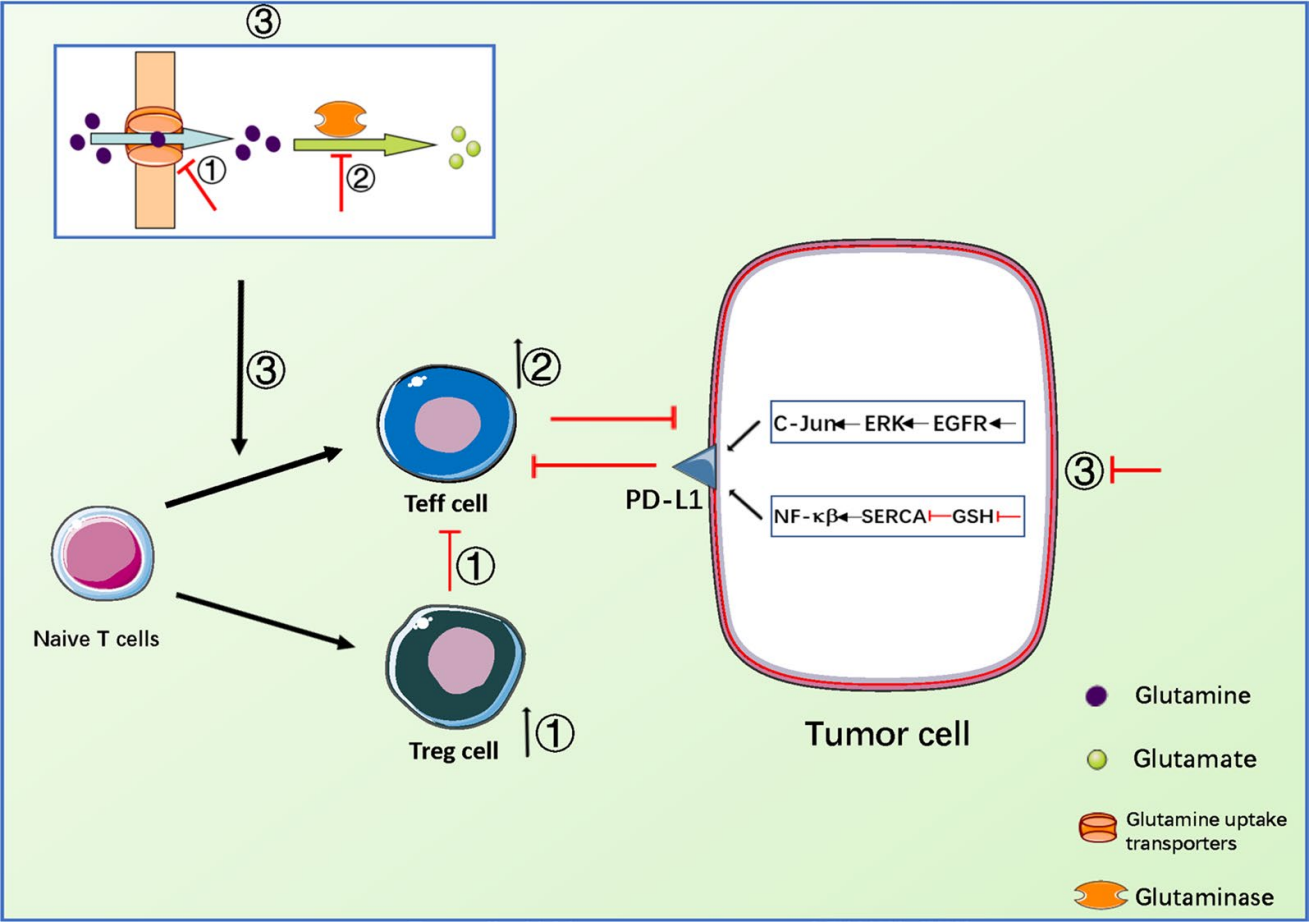
Reprogramming of glutamine metabolism in tumor cells and T cells and its impact on immune response. Inhibition of the glutamine transporter inhibits the differentiation of Teff cells while simultaneously promotes the differentiation of Treg cells (①). Inhibition of the GLS promotes the differentiation and effector function of Teff cells (②). Glutamine deprivation affects the differentiation of naive T cells, and up-regulates the expression of PD-L1 in tumor cells by activating the EGFR/ERK/C-Jun signaling pathway or reducing GSH levels, inhibiting SERCA activity, and then activating the NF-κB signaling pathway (③)

Glutamine metabolism maintains tumor survival and progression, and is very important for multiple biological processes such as nucleotide synthesis, amino acid production, protein glycosylation modification, extracellular matrix production, epigenetic modifications, maintenance of cellular redox balance, and autophagy [68]. In addition to the direct effects of altered glutamine consumption on the function of immune cells and glutamine metabolism in the TME, the functional changes in the tumor cells themselves also directly affects the anti-tumor response. For example, after glutamine deprivation in the culture medium, the renal cancer cell lines and bladder cancer cell lines up-regulated the expression of PD-L1 in tumor cells by activating the EGFR/ERK/C-Jun pathway, which is an important immunosuppressive molecule that binds to the PD-1 receptor on the surface of immune cells, inhibiting the anti-tumor immune response. Therefore, co-culture with peripheral blood T lymphocytes (PBTLs) may inhibit the production of IFN-γ from T cells, thereby inhibiting the anti-tumor immune response [69, 70].Interestingly, in another study, researchers found that restricting glutamine consumption by tumors up-regulated the expression of tumor PD-L1, by reducing the expression of GSH in the tumor cells. Mechanistically, glutamine is a major precursor for glutathione synthesis, therefore, limiting the utilization of glutamine by tumor cells leads to the reduction in GSH, inhibits the activity of sarcoplasmic reticulum Ca^2+^-ATPase (SERCA), and activates the NF-κB signaling pathway, thereby promoting the expression of tumor PD-L1 and inactivating the co-cultured T cells. In mouse models of tumor, targeting glutamine metabolism combined with monoclonal antibody against PD-L1 may further improve the anti-tumor immune response [71] (Fig. 2).

The unique metabolic properties of tumor cells are essential features that distinguish them from normal cells. However, the molecular mechanisms regulating glutamine metabolism in the tumor is not yet fully understood. Also, the molecular mechanisms regulating immune checkpoints such as PD-L1 and CD47 are still unclear. Moreover, little is known about the regulatory relationship between glutamine metabolism and immune checkpoint regulation. Some recent studies have shown that there were same regulatory molecules enabling the crosstalk between glutamine metabolism and the expression of immune checkpoint proteins. As previously highlighted, the proto-oncogene *Myc* has been shown to be critical for glutamine metabolism. MYC specifically activates the expression of glutamine transporter and glutaminase in tumors, thereby regulating the reprogramming of glutamine metabolism in tumors [30, 72, 73].At the same time, MYC also regulates the expression of PD-L1 and CD47 in the tumor cells. MYC expressed by the tumor cells not only regulates the tumor immune microenvironment by acting on innate and acquired immune cells and the secretion of cytokines, but also by direct action on the promoters of the genes encoding CD47 and PD-L1, which in turn regulates their mRNA and protein expression, eventually causing immunosuppression and tumor growth [74].The *Ras* oncogene promotes the reprogramming of glutamine metabolism in tumor cells by up-regulating the expression of glutaminase [75]. Mutation of the *K-Ras* gene activates the downstream signaling pathway involved in stabilizing the PD-L1 mRNA, thereby promoting PD-L1 protein synthesis by tumor cells and inhibiting the anti-tumor immune response [76]. In addition to *Myc* and *Ras*, HIF and *p53* have also been shown to be involved in the regulation of glutamine metabolism in tumors, as well as in the expression of immune checkpoints by tumor cells [77, 78]. Although many researches have confirmed that some of the same regulators are involved in mediating both the regulation of glutamine metabolism and immune checkpoints, these studies were independent and did not link glutamine metabolism with immune checkpoints expression. Therefore, whether these factors regulate the expression of immune checkpoints while regulating glutamine metabolism in tumors and thus affect the anti-tumor immune response needs to be further explored.

### Reprogramming of glutamine metabolism in immune cells and its impact on the immune response

Energy metabolism is an important basis for maintaining the activity and function of immune cells. In the process of immune cell activation, a large amount of energy and metabolic intermediates are required to meet the needs of macromolecule biosynthesis, so as to achieve cell proliferation, differentiation and effector functions. At the same time, the metabolic pathways of different types of immune cells during their activation, differentiation and proliferation are completely different from those in the resting state, suggesting the occurrence of “metabolic reprogramming” occurs [79]. In addition, changes in metabolic pathways further regulates the phenotype and function of immune cells, thereby affecting the body’s immune response. Glutamine is an important energy substrate for immune cells, and an important nitrogen and carbon donor for various biosynthetic precursors, and also plays a critical role in the activation and function of immune cells. Therefore, it is crucial to understand how changes in glutamine metabolism in immune cells affects their anti-tumor immune responses.

#### T cells

T cells are key players in the anti-tumor immune response. For example, activated CD8^+^T cells directly exert cytotoxic effects on tumor cells, and activated CD4^+^ T helper 1 (Th1) cells activate macrophages and NK cells by secreting IFN-γ, which promotes anti-tumor effects. While activated CD4^+^ T helper 2 (Th2) cells and regulatory T (Treg) cells promote tumor-induced immunosuppression, CD4^+^ T helper 17 (Th17) cells either support or inhibit tumor progression, depending on the context [80]. Usually in the resting state, the metabolic rate of the naive T cells is low; its demand for glutamine is low; and low levels of glutamine metabolism can maintain its survival [81].However, in the activated state, the Teff cells need to proliferate rapidly, thus increasing the intake of glutamine, which provides them with sufficient raw material for macromolecule synthesis, while promoting the secretion of cytokines [82].The decomposition of glutamine affects the differentiation of T cells. For example, when GlS1 is depleted, it promotes the differentiation and effector function of CD4^+^Th1 and CD8^+^ T cells by up-regulating the expression of the transcription factor T-bet, and inhibiting the mTORC1 and IL-2 signal transduction pathways, thereby inhibiting Th17 differentiation [83]. Additionally, the loss of GlS1 leads to α-kG deficiency, and impairs the differentiation of Th17 cells [84].Glutamine uptake transporters such as ASCT2, Solute carrier family 7, member 5 (SLC7A5) and Sodium-coupled Neutral Amino Acid Transporter (SNAT), when blocked, inhibit the differentiation of CD4^+^Th1 and Th17 cells [83], while SLC7A5-mediated glutamine uptake regulates the activation of c-MYC-dependent Teff cells. During glutamine deprivation, Teff cells show decreased c-MYC protein expression, growth restriction, and impaired immune function [85].In addition, glutamine deprivation promotes the differentiation of Treg cells through AMPK-mTORC1 signaling pathway, thereby reducing the immune function of Teff cells [86, 87].In summary, the reprogramming of glutamine metabolism in T cells regulates the differentiation and function of T cells from various aspects, thereby regulating the immune response of the body (Fig. 2).

#### Macrophages

**Fig. 3:**
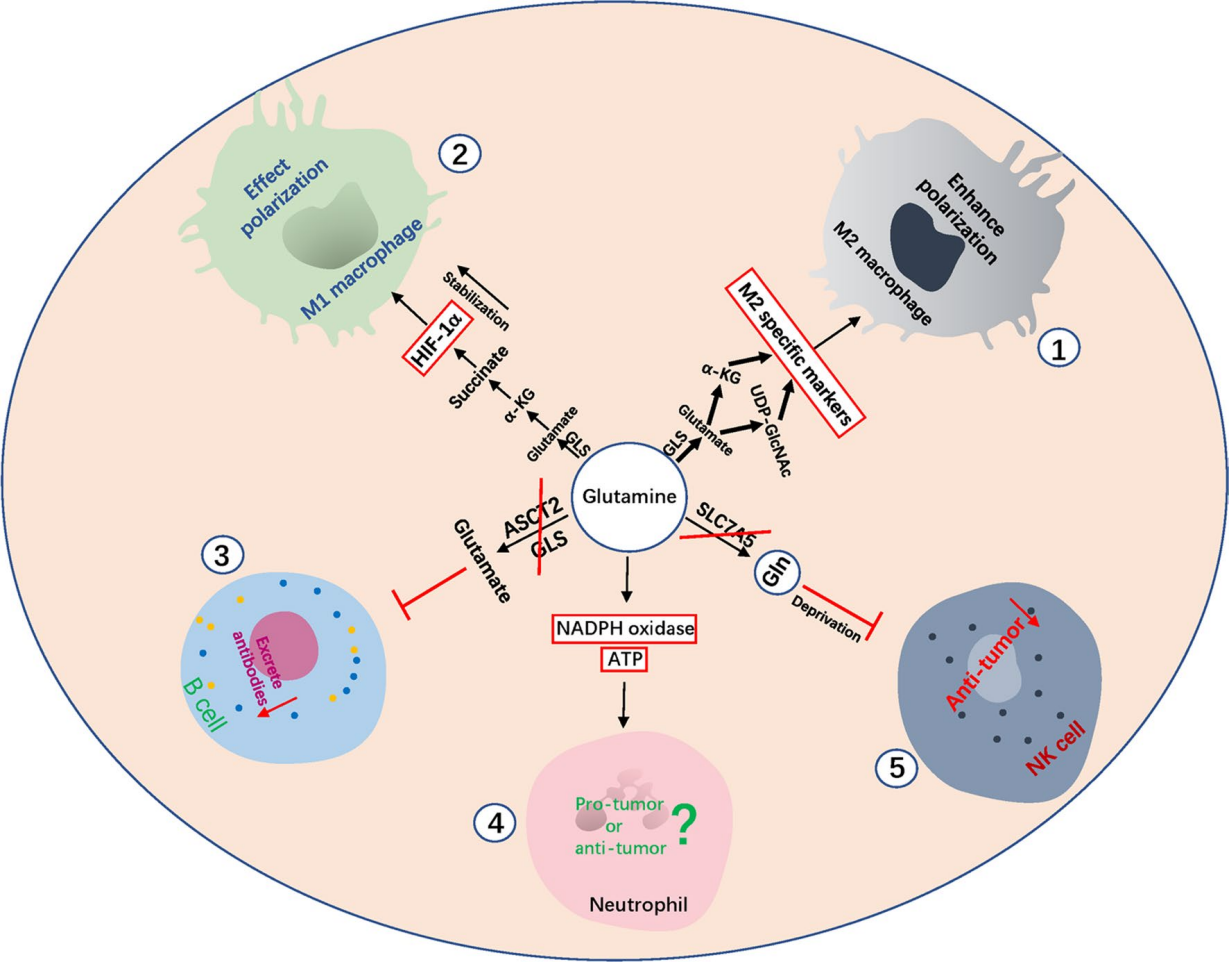
Impact of reprogramming of glutamine metabolism in immune cells on immune response. M2 macrophages consume more glutamine, and α-KG, a metabolite of glutamine metabolism, which promote the polarization of M2 macrophages. Glutamine metabolism in M2 macrophages is essential for supporting an active TCA cycle and UDP-GlcNAc synthesis. This provides the substrate for *N*-glycosylation, enabling the glycosylation of M2-marked proteins, and promoting the polarization of M2 macrophages (①). Glutamine metabolism in M1 macrophages promotes the accumulation of succinate by replenishing α-KG, further improving the stability of HIF-1α, which regulates the polarization of M1 macrophages (②). Inhibition of ASCT2 and GLS in B cells reduces the production of IgG and IgM antibodies (③). Glutamine regulates neutrophil function by generating ATP and regulating the expression of components of the NADPH oxidase complex, but its pro-tumor or anti-tumor effect is unknown (④). Suppression of glutamine metabolism in NK cells inhibits its anti-tumor function (⑤)

Macrophages are innate immune cells. Under the stimulation of lipopolysaccharide (LPS) and IFN-γ or IL-4, naive macrophages differentiate into M1 or M2 macrophages. M1 macrophages participate in the positive immune responses and play the role in immune surveillance by secreting inflammatory cytokines and chemokines, and are involved in professional antigen presentation. M2 macrophages possess a weak antigen-presenting ability, and secrete anti-inflammatory cytokines such as IL-10 or TGF-β, and down-regulate the immune response [88–90].Tumor-associated macrophages (TAMs) have been shown to be functionally plastic as a special type of macrophage, which are often described as M2-like population, but there is also evidence for the existence of M1-like population [91–93].In fact, in the early phase of tumor establishment, TAMs display an inflammatory phenotype, but an immunosuppressive phenotype is present at the later stages of tumor progression [94]. Glutamine metabolism plays an important role in the activation of macrophages, and there are inherent differences in the dependence of different macrophage subsets on glutamine. For example, early in vivo animal experiments showed that glutamine was essential for the production of cytokines (such as IL-1, IL-6, TNFα), antigen presentation, and phagocytic functions in murine macrophages [50]. Glutaminolysis affects the polarization of M1 macrophages. The uptake and metabolism of glutamine is elevated in LPS-activated M1 macrophages, and the replenishment of α-KG by glutamine metabolism further promotes the accumulation of succinate, improving the stability of HIF-1α, which in turn drives the production of pro-inflammatory cytokines (such as IL-1) [95, 96]. M2 macrophages consume more glutamine than M1 and naive macrophages, and usually glutamine accumulates in M2 macrophages and promotes its polarization. Part of the reason for this differential effect is that the metabolite of glutamine, α-kG, alters gene expression programs that support an anti-inflammatory M2-like state. Additionally, the expression of glutamine synthase (GS) is low in M1 macrophages, but high in M2 macrophages [58, 97, 98]. Recent studies have shown that in IL-4-induced M2 macrophages, glutamine is used to support active TCA cycle, and HBP. The HBP pathway produces UDP-GlcNAc, which acts as a substrate for N-glycosylation of M2-marked proteins, such as the N-glycosylation receptor CD206, as well as KLF4, CCL22, and IRF4, thereby promoting the polarization of M2 macrophages [96]. Supporting these observations, TAMs from Lewis lung cancer (M2 phenotype) was reported to express higher levels of the glutamine metabolizing enzymes, transaminase and glutamine synthetase. However, whether and how glutamine metabolism regulates the tumor-promoting function of TAMs remains to be further demonstrated [99]. Taken together, glutamine metabolism is involved in the polarization of M1 and M2 macrophages. Since M2 macrophages consume more glutamine than M1 macrophages, in the TME, it is unclear whether inhibiting the anti-tumor immune response from M2 macrophages polarization would be greater than the effect of enhancing the anti-tumor immune response from the M1 macrophages. Or is there a homeostasis between the M1 and M2 states. Furthermore, it is not known whether glutamine metabolism affects the differentiation of naive macrophages. All the above factors would determine whether the glutamine metabolism in macrophages promotes or inhibits the anti-tumor immune response (Fig. 3).

#### Other immune cells

B cells modulate the function of myeloid cells to support tumor progression, by producing antibodies and immune complexes [100, 101]. Glutamine is essential for the survival of B cells in hypoxic environments [102], and also promotes the differentiation of human B cells into plasma cells and lymphocytes [57]. In addition, antibody production by B cells depends on the breakdown of glutamine. When the expression of ASCT2 and GLS are inhibited, the production of IgG and IgM antibodies is reduced [103]. Neutrophils are normally recruited by chemokines released by tumors, and tumor-associated neutrophils (TANs) promote CD8^+^T cells responses and anti-tumor activity in the absence of tumor-derived TGF-β, whereas in the presence of TGF-β promotes the tumor-promoting activity of CD8^+^T cells [104]. Neutrophils consume glutamine at the highest rates relative to other leukocytes, such as macrophages and lymphocytes [105, 106]. Glutamine enhances superoxide production in neutrophils by generating ATP and regulates the expression of components of the NADPH oxidase complex [107]. Furthermore, glutamine plays an important role in preventing adrenaline induced changes in NADPH oxidase and superoxide production in neutrophils [108]. NADPH oxidase is essential for neutrophil function, as neutrophils use extracellular traps (NETs) to perform their functions, and the action of NETs requires the activation of NADPH oxidase [109]. Therefore, glutamine metabolism is crucial for the function of neutrophils, but its pro-tumor or anti-tumor effects need to be further explored. Natural killer (NK) cells play an integral role in activating anti-tumor T cell responses and killing tumor cells by producing IFN-γ and cytotoxic molecules such as granzyme. Glutamine uptake mediated by the glutamine transporter SLC7A5, regulates the activation of c-MYC-dependent NK cells. When glutamine is deprived, NK cells exhibit reduced expression of c-MYC protein, growth restriction, and impaired immune function, while inhibition of glutamine breakdown has no effect on NK cells [85]. In addition to the immune cells discussed above, other immune cells have also been shown to play an important role in the anti-tumor immune response. However, the regulation of glutamine metabolism in these immune cells in the TME remains unknown, and further studies are needed to demonstrate its role in the body’s immune response (Fig. 3).

### Glutamine metabolism inhibitors and their effect on immune response

Glutamine in the TME not just meets the metabolic needs of the rapidly proliferating tumor cells, but also does the same for the different types of immune cells. As mentioned above, the differential impact of glutamine metabolism on different types of cells in the TME would eventually determine the outcome of targeting glutamine metabolism, and its effect on tumor suppression and anti-tumor immune response. The current drugs targeting glutamine metabolism are mainly classified into three categories, namely, glutamine antimetabolites, glutaminase inhibitors and glutamine uptake inhibitors. Several recent studies have demonstrated that the above three classes of glutamine metabolism inhibitors positively impact the function of different immune cells in the TME, while inhibiting tumor cell proliferation.

#### Effects of glutamine antimetabolites on immune response

**Fig. 4:**
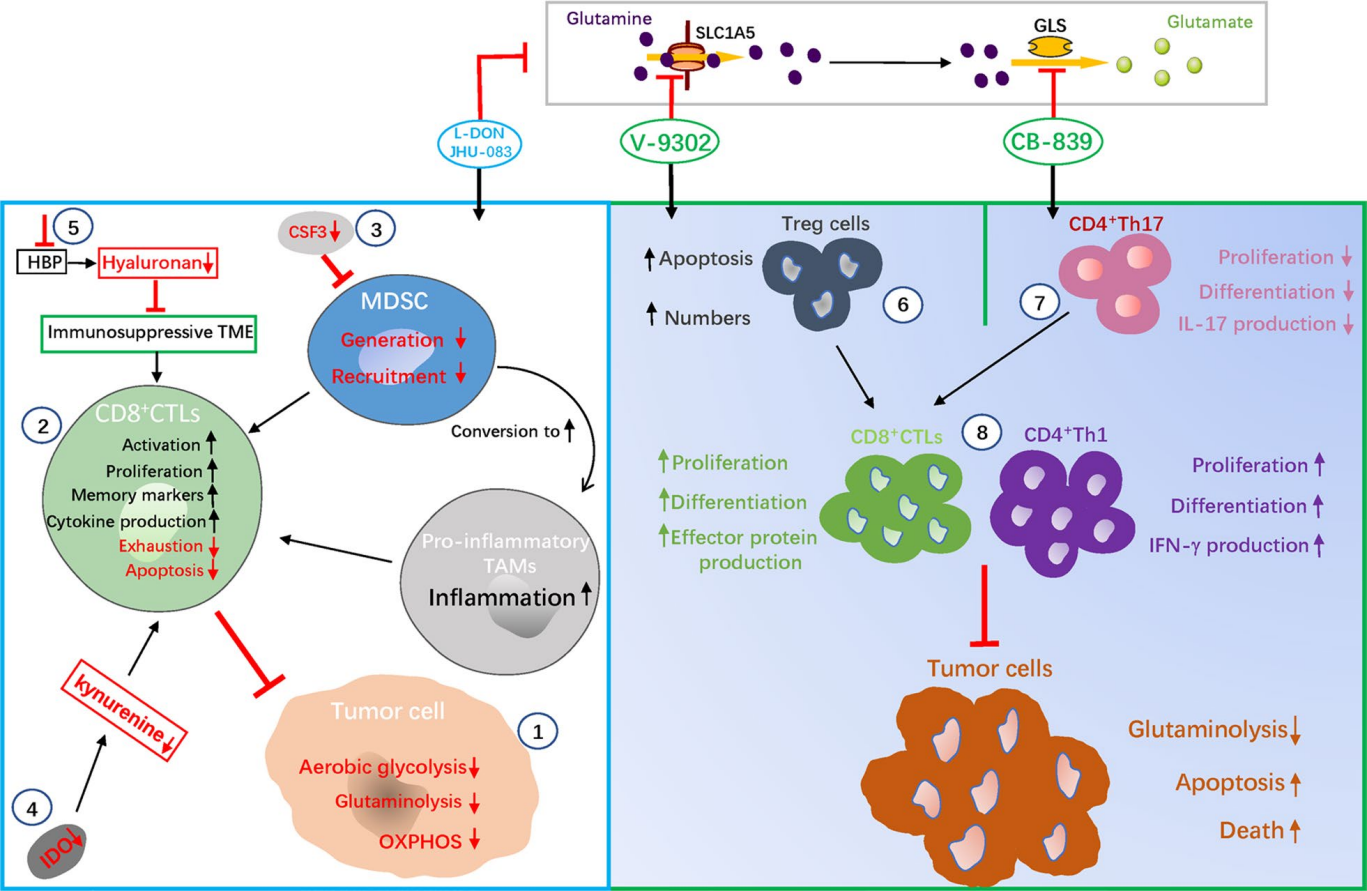
Effects of glutamine metabolism inhibitors on immune response. Glutamine antimetabolites L-DON and JHU-083 can inhibit glutamine metabolism, glycolysis, and OXPHOS in tumor cells, and comprehensively disintegrate the energy metabolism of tumors (①). Glutamine antimetabolites directly modulate the metabolism of CD8^+^CTLs to promote a long-lasting, activated, memory-like phenotype; enhance cytokine production; and inhibit exhaustion and apoptosis (②). Glutamine antimetabolites inhibit the generation and recruitment of MDSCs and induce the differentiation of MDSCs and TAMs into pro-inflammatory TAMs by suppressing the secretion of CSF3 (③). Glutamine antimetabolites inhibit the expression of IDO in tumor and myeloid derived cells; reduce the levels of kynurenine; and enhance anti-tumor immune response (④). Glutamine antimetabolites inhibit the HBP metabolic pathway; decrease the levels of hyaluronan; change the mechanical properties of the ECM; improve the immunosuppressive TME and enhance the anti-tumor immune response (⑤). Glutamine uptake inhibitor V-9302 has distinct effects on the differentiation of T cell subsets, favoring CD4^+^Th1 and CD8^+^CTL but reducing the levels of Treg cells (⑥, ⑧). Glutaminase inhibitor CB-839 enhances the activation of CD4 ^+^ Th1 and CD8^ +^ CTL but suppress the differentiation of CD4^+^Th17 cells (⑦, ⑧)

6-Diazo-5-oxo-L-norleucine (L-DON), as a first-generation glutamine antimetabolite, inhibits all the glutamine-utilizing enzymes. Although it promotes a strong anti-tumor effect, systemic toxicity limits its clinical application [110–112]. To address this problem, researchers developed JHU-083, a prodrug form of L-DON, which is selectively activated to L-DON after entering the TME, thus reducing its systemic toxicity and improving its anti-tumor immune response [113, 114]. In syngeneic mouse models treated with JHU-083, the metabolic activity of the tumor was extensively suppressed, while hypoxia was mitigated and the levels of glutamine and glucose in the TME were increased. When combined with anti-PD-1 monoclonal antibody, the complete response rates approached 100% as compared to treatment with immunotherapy alone. Therefore, it is proposed that JHU-083 may not have negative effects on immune cells, but may enhance the function of immune cells. Subsequent metabolic flux analysis showed that in vitro treatment with L-DON altered CD8^+^T cells towards an activated, long-lived, and memory-like state. Correspondingly, in vivo treatment of tumors with JHU-083 had an increased the number of CD8^+^tumor-infiltrating lymphocytes (TILs), and their transcriptional program showed an enhanced proliferative capacity and anti-cancer activity, and the expression of genes related to long-term memory CD8^+^T cells was up-regulated, while the expression of genes related to apoptosis was significantly down-regulated. Mechanistically, L-DON and JHU-083 suppressed tumor glutamine metabolism by inhibiting all the glutamine-utilizing enzymes, and simultaneously suppressed tumor glycolysis by activating AMP Kinase (AMPK) and inhibiting the expression of c-MYC [114, 115]. AMPK and c-MYC are recognized as key regulators of glycolytic flux [116–118]. Furthermore, OXPHOS in tumor cells was also suppressed due to the absence of alternative fuels as carbon sources for the TCA cycle [114]. Overall, due to the lack of plasticity in the interdependence of glycolysis, OXPHOS and glutamine metabolism in tumor cells, extensive inhibition of glutamine metabolism in tumor cells inhibits their glycolysis and OXPHOS, thereby comprehensively disintegrating the energy metabolism in tumor cells [114]. Although glutamine metabolism and glycolysis are inhibited in CD8^+^T cells, their OXPHOS is up-regulated, and the extracellular acetate is used as an alternative fuel to generate ATP, further activating the anti-tumor effects of CD8^+^T cells [114]. In addition to CD8^+^T cells, JHU-083 also affects the immune function of myeloid-derived suppressive cells in the TME. Generally, myeloid-derived suppressor cells (MDSCs) and TAMs in the TME inhibit the anti-tumor immune response. In tumor-bearing mouse models treated with JHU-083, tumor growth was found to be suppressed and the generation and recruitment of MDSCs was also markedly inhibited. Mechanistically, targeting tumor glutamine metabolism in the tumors promoted a decrease in CSF3secretion, and promoted the differentiation of MDSCs and TAMs into pro-inflammatory TAMs. Additionally, blocking glutamine metabolism also inhibited the expression of IDO in the tumor and myeloid derived cells, resulting in a significant reduction in the levels of kynurenine, further enhancing the anti-tumor immune response [119]. Interestingly, L-DON also enhanced the anti-tumor immune response by affecting the mechanical properties of the tumor extracellular matrix (ECM), which is responsible for the formation of the immunosuppressive TME. Hyaluronan is the main component of the ECM, and its precursor is synthesized by the HBP, and L-DON inhibits glutamine-fructose aminotransferase 1 (GFPT1), the rate-limiting enzyme in the HBP, resulting in decreased hyaluronan synthesis, affecting the mechanical properties of the ECM in the TME and enhancing the infiltration of CD8^+^T cells and the anti-tumor immune response [120]. In conclusion, glutamine antimetabolites effectively inhibit tumor growth while improving the anti-tumor immune response through multiple mechanisms, revealing the close interaction between glutamine metabolism and immune response in the TME (Fig. 4).

#### Effects of glutaminase inhibitors on immune response

GLS is highly expressed in diverse malignancies and is essential for their survival, and tumor-targeted drugs targeting GLS have been extensively studied. BPTES and 968, are the two major classes of GLS inhibitors that have been shown to have anti-tumor activity [42, 121]. CB-839, a BPTES-based allosteric GLS inhibitor, with a better oral bioavailability and stronger inhibitory activity, is being tested in clinical trials [122]. Previous research has shown that BPTES treatment promoted pro-inflammatory M1-like activation of macrophages, thereby enhancing the body’s anti-tumor immune response [58]. However, BPTES treatment also promoted the upregulation of PD-L1 expression, which inhibited the anti-tumor function of immune cells upon PD-L1 binding to the PD-1 receptor on the surface of immune cells. Therefore, there is the possibility of immune escape after treating tumor cells with BPTES [71]. CB-839 has varied effects on the function of different types of T cells, thereby affecting the immune response. In vitro experiments showed that CB-839 treatment promoted the differentiation of CD4^+^Th1 cells and CD8^+^ cytotoxic T lymphocytes (CTLs) and induced both the cell types to secrete more cytokines. However, treatment of Th17 cells with CB-839 inhibited their differentiation, function, and cytokine production, and eventually suppressed their expansion. The reason for these opposing outcomes may be due to differences in the epigenetic response of each T cell subset to α-KG depletion after GLS blockade [65, 83]. Importantly, there is temporal heterogeneity in the response of Teff cells to CB-839 treatment, and others have shown that short-term ex vivo treatment of Teff cells with CB-839 enhanced the subsequent anti-tumor function of Th1 cells and CD8^+^ CTLs in vivo. However, it did not lead to a long-term effect. In addition, transient exposure to CB-839 in vitro improved the function of chimeric antigen receptor (CAR) T cells, in a mouse model receiving CAR-T cell immunotherapy for a limited time [83]. Therefore, the glutaminase inhibitor CB-839 enhances the function of Teff cells while inhibiting the function of Treg cells, thereby enhancing the anti-tumor immune response (Fig. 4).

#### Effects of glutamine uptake inhibitors on immune response

The glutamine transporter SLC1A5, is frequently up-regulated in the tumor cells, and its overexpression is associated with a poor prognosis in cancer patients [30, 123]. V-9302, an inhibitor targeting SLC1A5, significantly inhibits tumor cells proliferation in vitro, and suppresses tumor growth in mouse models, and also modulates the anti-tumor immune response [124]. In a spontaneous mouse model of TNBC, researchers found that V-9302 selectively blocked glutamine uptake in TNBC cells to inhibit tumor growth, but did not inhibit the T cells. Also, T cell activation was enhanced, and the levels of TILs were significantly increased, the anti-tumor function of CD8^+^T cells and CD4^+^Th1 cells were enhanced, and supported the transition of CD8^+^T cells to long-term memory cells, while the levels of Treg cells were reduced. Mechanistically, V-9302 sustained glutamine uptake by CD8^+^T cells, by promoting the compensatory upregulation of the glutamine transporter ATB^0,+^/*Slc6a14*, in CD8^+^T cells, to support de novo glutathione synthesis and improve redox balance in T cells. However, such compensatory upregulation of glutamine transport was not found in V-9302-treated TNBC cells [17]. Meanwhile, in human breast cancer cell lines, researchers found that V-9302 enhanced anti-tumor response by promoting ROS production, which induced the autophagic degradation of B7 homology 3 (B7H3). B7H3 is considered to act as an immune checkpoint ligand that contributes to immune escape. In mouse models of breast cancer, V-9302 treatment significantly increased autophagosome formation and decreased the expression of B7H3 in tumor cells, and enhanced CD8^+^ T cell infiltration and activation. The combination of V-9302 and anti-PD-1 antibody showed a greater anti-tumor effect than either of the single treatments [125, 126]. Consistent with BPTES, tumor cells treated with V-9302 up-regulated the expression of PD-L1, Thus, there is the possibility of immune escape after treating tumor cells with V-9302, and the combined targeting of glutamine metabolism and PD-L1 showed a greater anti-tumor efficacy in mouse tumor models [71]. In conclusion, V-9302, a glutamine uptake inhibitor, not just enhanced the anti-tumor function of CD8^+^T cells, but also induced immune escape by up-regulating the expression of PD-L1 in tumor cells, indicating that V-9302 had a complex effect on the immune response (Fig. 4).

## Conclusions

The critical role of glutamine in energy generation and macromolecule synthesis underlies its importance in tumor progression and immune response. Therefore, further studies exploring the role of glutamine metabolism in the tumors and immune cells would help us to develop therapeutic strategies for targeting glutamine metabolism in the TME for cancer therapy. In fact, both tumor cells and immune cells greatly depend on the availability of glutamine to survive, proliferate, and function. Reprogramming of glutamine metabolism in the tumor cells to their biosynthetic and energy requirements to support their rapid proliferation and survival in a hypoxic TME. And reprogramming of glutamine metabolism in the immune cells maintains their survival while modulating their phenotypes and function, contributing to their pro-tumorigenic or anti-tumorigenic functions. These observations suggest that: ① there is an association in glutamine metabolism between tumor cells and immune cells in the TME, ② reprogramming of glutamine metabolism in tumor cells and immune cells is closely related to the immune response, and ③ the different rate of glutamine metabolism in the tumor and immune cells determines their differential response to glutamine metabolism inhibitors. For example, there may be a competition for glutamine between immune and tumor cells, and cell-programmed glutamine partitioning may happen between these cells in the TME. Glutamine metabolism affects immune response by regulating the differentiation and activity of Teff cells, Treg cells, and macrophages, and the expression of immune checkpoint proteins in tumor cells. Also, different types of glutamine metabolism inhibitors have been reported to have varying effects on different immune cells, while inhibiting the proliferation of tumor cells. Together, the above factors determine how glutamine metabolism in the TME affects the immune response, eventually causing tumor progression or suppression.

Combination therapy with immunotherapeutic agents and drugs targeting tumor metabolism are a major focus of current cancer research. Studies have reported a synergistic effect of targeting glutamine metabolism and anti-tumor immunity. However, the current clinical trials related to the above combination therapy have not achieved satisfactory results [127]. The reasons for this may include the inherent heterogeneity in glutamine metabolism in the tumor and immune cells, and the intricate effects of glutamine metabolism on immune responses. As discussed in our paper, glutamine uptake inhibitors not only activate the function of Teff cells in the TME, but also up-regulate the expression of PD-L1 in tumor cells, which may inhibit the function of Teff cells. This indicates the complexity of the effects of glutamine metabolism on immune responses. Although the current studies have confirmed that various types of glutamine metabolism inhibitors not just inhibit tumor proliferation effectively, but also have a positive impact on the anti-tumor function of immune cells. However, one needs to evaluate whether they also modulate the expression of immune checkpoint proteins in tumor cells and contribute to immune escape. Therefore, a deeper investigation of glutamine metabolism in tumor cells and immune cells, and the crosstalk between these cells would help us understand the mechanisms associated with immune evasion, and the glutamine requirements by immune cells, which would be crucial to fully realize the effect of combination therapy.

## Data Availability

Data sharing is not applicable to this article as no new data were created or analyzed in this study.

## Abbreviations

TME: Tumor microenvironment
ROS: Reactive Oxygen species
NK cells: Natural killer cells
DCs: Dendritic cells
GLS: Glutaminase
α-KG: α-Ketoglutarate
TCA: Tricarboxylic acid cycle
HIF: Hypoxia-inducible factor
LDHA: Lactate dehydrogenase A
GSH: Glutathione
NADPH: Nicotinamide adenine dinucleotide phosphate
NF-κB: Nuclear factor-kappa B
IFN-γ: γ-Interferon
CAFs: Cancer-associated fibroblasts
PBTLs: Peripheral blood T lymphocytes
SERCA: Sarcoplasmic reticulum Ca^2^^+^-ATPase
Th1: CD4^+^ T helper 1
Th2: CD4^+^ T helper 2
Treg: Regulator T
Th17: CD4^+^ T helper 17
SLC1A5: Solute carrier family 1 neutral amino acid transporter member 5
ASCT2: Alanine, serine, cysteine-preferring transporter 2
SLC7A5: Solute carrier family 7, member 5
SNAT: Sodium-coupled neutral amino acid transporter
LPS: Lipopolysaccharide
TAMs: Tumor-associated macrophages
GS: Glutamine synthase
HBP: Hexosamine biosynthetic pathway
TANs: Tumor-associated neutrophils
NETs: Neutrophil extracellular traps
L-DON: 6-Diazo-5-oxo-L-norleucine
AMPK: AMP kinase
TILs: CD8^+^tumor-infiltrating lymphocytes
OXPHOS: Oxidative phosphorylation
MDSCs: Myeloid-derived suppressor cells
ECM: Extracellular matrix
GFPT1: Glutamine-fructose aminotransferase 1
CTLs: Cytotoxic T lymphocytes
CAR: Chimeric antigen receptor
TNBC: Triple-negative breast cancer
B7H3: B7 homology 3
